# Supplementary material for: Post-processing methods for delay embedding and feature scaling of reservoir computers
Source: Commun Eng. 2025 Jan 27;4:10. doi: 10.1038/s44172-024-00330-0 (PMC11772693; doi:10.1038/s44172-024-00330-0)
Supplement: Supplementary file 2 — Supplementary material [file 44172_2024_330_MOESM2_ESM.pdf]

Post-processing methods for delay-embedding and feature scaling of reservoir computers

Jonnel Jaurigue[1],* Joshua Robertson[2], Antonio Hurtado[2], Lina Jaurigue[1], and Kathy Lüdge[1]
[1] *Technische Universität Ilmenau, Institut für Physik, Weimarer Straße 25, 98693 Ilmenau, Germany and*
[2] *University of Strathclyde, Institute of Photonics, SUPA Department of Physics, 99 George Street, G1 1RD, Glasgow, UK*

SUPPLEMENTARY MATERIAL

Supplementary Note 1: Additional simulated reservoir computer results of Lorenz x-to-z cross-prediction

Suppl. Figure 1 graphs the timeseries of predicted Lorenz z-coordinates $\hat{\boldsymbol{y}}$ versus target $\boldsymbol{y}$, for increasing N replicas. We see better overlap of the prediction to target (grey dotted-line) with increasing N replicas, corresponding to the improved NRMSE.

Suppl. Figure 2 demonstrates superior performance (dark teal/black) at total or near total recall recall $J \approx n$ even when the readout node dimension is not rescaled, but kept at $2n$.

Suppl. Figure 3 illustrates the scan of increasing recall J with increasing uniform-timeshift d_2. These data are derived from the same realisation set used for main article Figure 4c, but map covariance rank instead of NRMSE. We see that covariance rank of the uniform-timeshift state matrix $\boldsymbol{S}^\frown \boldsymbol{S}_{\boldsymbol{d}_2}$ generally increases (dark teal/black) with uniform-timeshift d_2, and that larger covariance rank is not correlated with zones of superior performance shown in Figure 4c. This finding corroborates main article Figure 3d, where covariance rank of the random-timeshifts state matrix $\boldsymbol{S}_{\boldsymbol{r}^1}$ monotonically increased with max-random-timeshift R.

Suppl. Table I compares the performance of multi-random-timeshifts state matrices $\boldsymbol{S}_{\boldsymbol{r}^1}{}^\frown ... {}^\frown \boldsymbol{S}_{\boldsymbol{r}^N}$ derived from simulated reservoir computers of different size. The "clamped reservoir of n nodes, total recall $J = n$" results are derived from a clamped reservoir where all n nodes are reiterated up to N times. The "rescaled reservoir of Nn nodes, null recall $J = 0$" results are derived from rescaled reservoirs, so that the Nn features for increasing N replicas had to be sampled from larger reservoirs upscaled to node dimension Nn. We observed that performance of the clamped reservoir at total recall $J = n$ performed just as well as the correspondingly upscaled reservoir at null recall $J = 0$.

Supplementary Note 2: Simulated reservoir computer results of Lorenz x-to-x 1-step-ahead forecasting

Suppl. Figure 4 illustrates that when we changed the task from Lorenz x-to-z cross-prediction to x-to-x 1-step-ahead forecasting, optimal performance was achieved when max-random-timeshift $R = 0$ (teal line). At this optimal max-random-timeshift R configuration the covariance rank (maroon line) of the state matrix is minimal. Thus, a large covariance rank may not guarantee good performance, and the assumption that a state matrix with a larger covariance rank generally outperforms a state matrix of a smaller covariance rank[1–4] is incomplete.

Supplementary Note 3: Simulated reservoir computer results of Mackey-Glass P-to-P 10-step-ahead forecasting

The simulated reservoir training models established on the Lorenz attractor were corroborated with the Mackey-Glass P-to-P 10-step-ahead forecasting task.

The results of Mackey-Glass P-to-P 10-step-ahead forecasting are summarised on Suppl. Table II. As expected, increasing the number of replicas N improved performance for uniform-timeshifting as well as random-timeshifting. The optimal uniform-timeshift d_2 changes with N, indicating that uniform-timeshifts $\{d_1, ...d_N\}$ must be re-optimised with changing replicas number N. Main article Figure 6d illustrates zones of optimal uniform-timeshift d_2 and d_3 configurations (dark teal/black area). Suppl. Figure 5 illustrates the expected trend where optimal max-random-timeshift R (maroon dots) increases with feature dimension.

Recalling past states was shown to improve performance while reducing reservoir node dimension. Suppl. Figure 6 demonstrates this phenomenon, with scans for recall J versus uniform-timeshifts d_2 and d_3. We see that performance improves as recall $J \approx n$ (dark teal/black). The node dimension is subsequently reduced from $3n$ to n.

Suppl. Figures 7 and 8 illustrate that changing the internal time-delay τ affects reservoir memory, which in turn affects the optimal uniform-timeshift d_2. We see performance improving at resonances (teal-coloured streaks) of the delay and the uniform-timeshift d_2, as

* Email address: jonnel-anthony.jaurigue@tu-ilmenau.de

we go from null recall $J = 0$ (Suppl. Figure 7, lesser performance at resonance) to total recall $J = n$ (Suppl. Figure 8, superior performance at resonance). The improved performance at resonance is opposite to previous observations[5] and remains to be understood.

Supplementary Note 4: Simulated reservoir computer results of Rössler x-to-z cross-prediction

The simulated reservoir training models established on the Lorenz attractor were corroborated with the Rössler x-to-z cross-prediction task.

Rössler attractor driving input-series $\boldsymbol{x}$ was the Rössler x-coordinate defined by

$$\frac{\mathrm{d}x}{\mathrm{d}t} = -y - z,$$
$$\frac{\mathrm{d}y}{\mathrm{d}t} = x + ay,$$
$$\frac{\mathrm{d}z}{\mathrm{d}t} = b + z(x - c)$$

and cross-prediction target-series $\boldsymbol{y}$ was the Rössler z-coordinate[6]. System parameters for the Rössler attractor are given in Suppl. Table III.

The results of Rössler x-to-z cross-prediction are summarised on Suppl. Table IV. As expected, increasing the number of replicas N improved performance for uniform-timeshifting as well as random-timeshifting. The optimal uniform-timeshift d_2 changes with N, indicating that uniform-timeshifts $\{d_1, ...d_N\}$ must be re-optimised with changing replicas number N. Suppl. Figure 9 illustrates zones of optimal uniform-timeshift d_2 and d_3 configurations (dark teal/black area). Suppl. Figure 10 illustrates the expected trend where optimal max-random-timeshift R (maroon dots) increases with feature dimension.

Recalling past states was shown to improve performance while reducing reservoir node dimension. Suppl. Figure 11 demonstrates this phenomenon, with scans for recall J versus uniform-timeshifts d_2 and d_3. We see that performance improves as recall $J \approx n$ (dark teal/black). The node dimension is subsequently reduced from $3n$ to n.

The simulated reservoir computer parameters were optimised on the Lorenz attractor. Given the similarities between the Lorenz and Rössler attractors [6] we observed better performance on the Rössler attractor compared to the Mackey-Glass attractor. Regularisation λ values optimised for the Lorenz attractor remained consistent for the other attractors, likely due to the conserved reservoir computer parameters.

Supplementary Note 5: Physical reservoir computer results of Mackey-Glass P-to-P 1-step-ahead forecasting

Physical reservoir results for Mackey-Glass P-to-P 1-step-ahead forecasting task are summarised on Suppl. Table V. Training on the base state matrix $\boldsymbol{S}$ (uniform-timeshifting $N = 1$) corroborates with previously published results for this task[7].

SUPPLEMENTARY MATERIAL REFERENCES

[1] T. L. Carroll and L. M. Pecora, Network structure effects in reservoir computers, Chaos **29**, 083130 (2019).

[2] T. L. Carroll and J. D. Hart, Time shifts to reduce the size of reservoir computers, Chaos **32**, 083122 (2022).

[3] L. Storm, K. Gustavsson, and B. Mehlig, Constraints on parameter choices for successful time-series prediction with echo-state networks, Mach. Learn.: Sci. Technol. **3**, 045021 (2022).

[4] J. D. Hart, F. Sorrentino, and T. L. Carroll, Time-shift selection for reservoir computing using a rank-revealing QR algorithm, Chaos **33**, 043133 (2023).

[5] F. Köster, S. Yanchuk, and K. Lüdge, Insight into delay based reservoir computing via eigenvalue analysis, J. Phys. Photonics **3**, 024011 (2021).

[6] O. E. Rössler, An equation for continuous chaos, Phys. Lett. A **57**, 397 (1976).

[7] J. Bueno, J. Robertson, M. Hejda, and A. Hurtado, Comprehensive performance analysis of a vcsel-based photonic reservoir computer, IEEE Photon. Technol. Lett. **33**, 920 (2021).

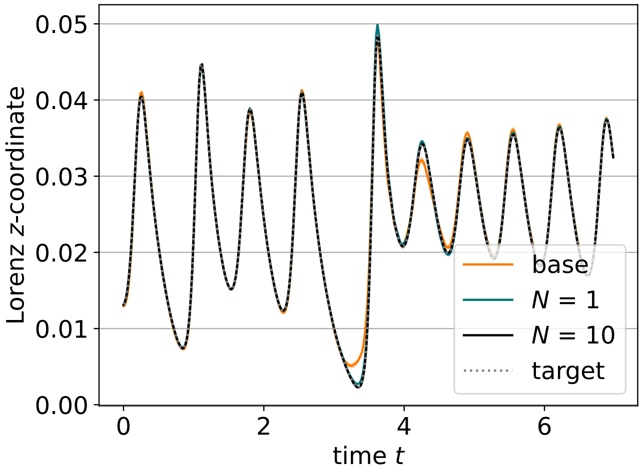

Suppl. Figure 1. Timeseries of Lorenz x-to-z cross-prediction on the simulated reservoir computer. Lorenz z-coordinates over time t, showing base (orange line) and random-timeshifting at replica number $N = 1$ (teal line) or $N = 10$ (black line) predictions of the z-coordinate, compared to the target (grey dotted-line). Note the overlap between $N = 10$ and target. Shaded area around each prediction line indicates the median absolute deviation of the realisation set.

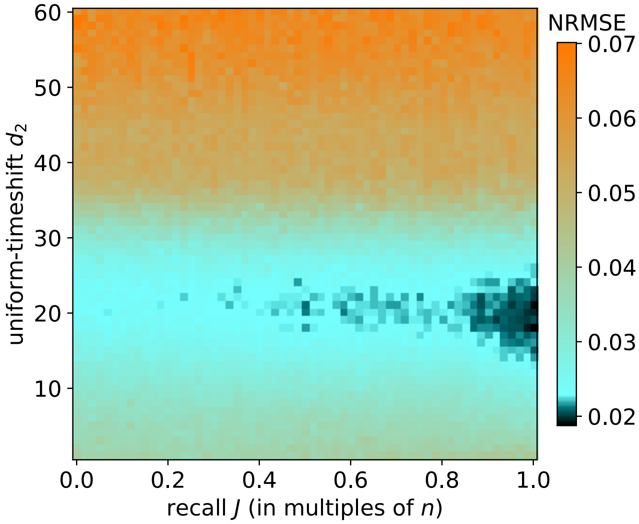

Suppl. Figure 2. Recall implementation on the simulated reservoir computer, Lorenz x-to-z cross-prediction. Scan of recall J versus uniform-timeshift d_2, for testing error at $O = 2n$ features on an unscaled reservoir fixed at $2n$ nodes. NRMSE; normalised root mean square error.

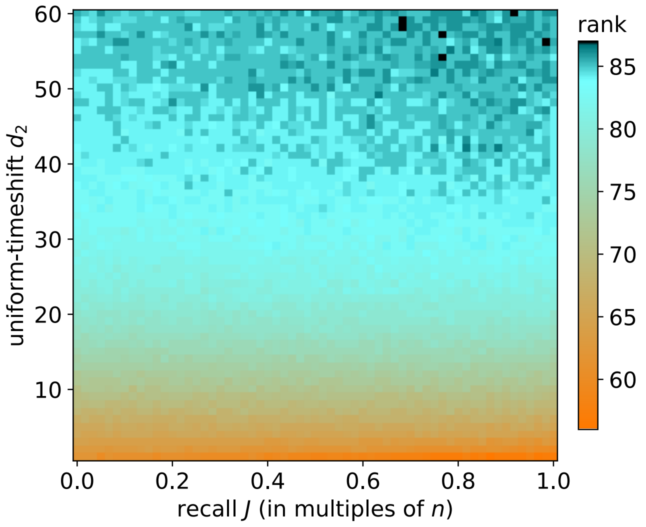

Suppl. Figure 3. Recall implementation on the simulated reservoir computer, Lorenz x-to-z cross-prediction. Scan of recall J versus uniform-timeshift d_2, for covariance rank at $O = 2n$ features. Graph corresponds to main article Figure 4c.

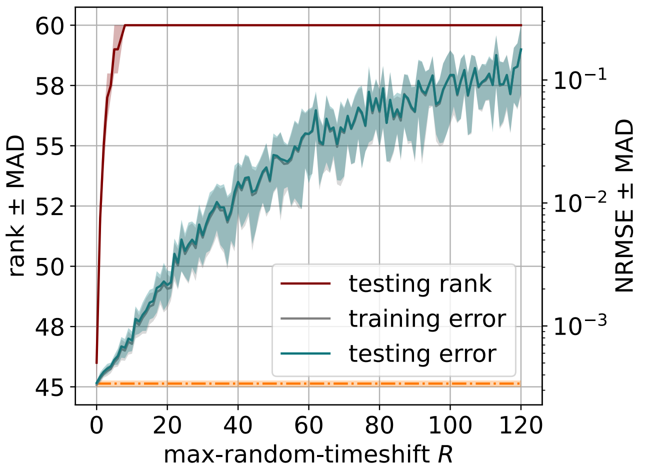

Suppl. Figure 4. Random-timeshifting on the simulated reservoir, Lorenz x-to-x 1-step-ahead forecasting. Scan of max-random-timeshift R for covariance rank (maroon line), training error (grey line) and testing error (teal line) at $N = 1$ replica and $O = n$ features. Orange dashed-line indicates result of training on the base state matrix $\boldsymbol{S}$. Shaded area around each line indicates the median absolute deviation of the realisation set. NRMSE; normalised root mean square error. MAD; median absolute deviation.

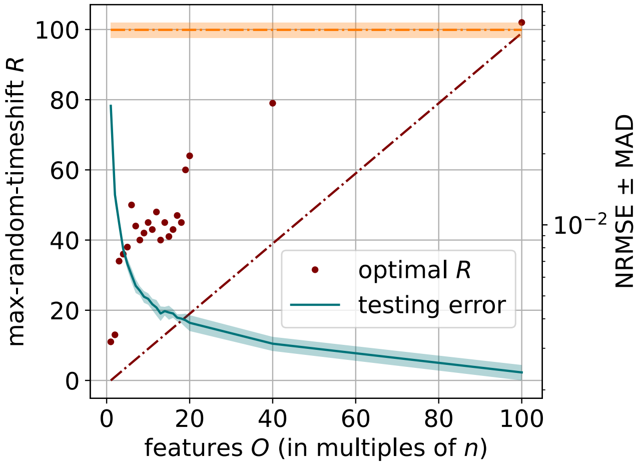

Suppl. Figure 5. Multi-random-timeshifting on the simulated reservoir computer, Mackey-Glass P-to-P 10-step-ahead forecasting. Scan of feature dimension O (up to $O = 100n$ features) versus max-random-timeshift R, for testing error (teal line). Maroon dots indicate optimal max-timeshift R at feature dimension O. Maroon dashed-line indicates the minimum value that the max-random-timeshift R may take at a given feature dimension. Orange dashed-line indicates result of training on the base state matrix $\boldsymbol{S}$. Shaded area around each line indicates the median absolute deviation of the realisation set. NRMSE; normalised root mean square error. MAD; median absolute deviation.

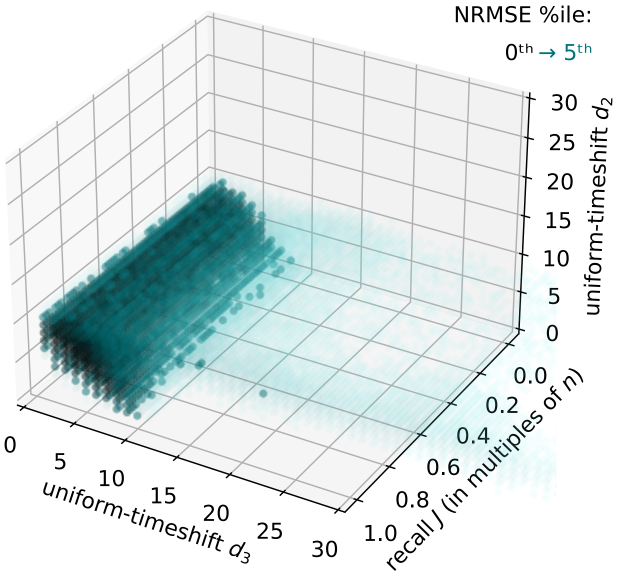

Suppl. Figure 6. Recall implementation on the simulated reservoir computer, Mackey-Glass P-to-P 10-step-ahead forecasting. Scan of recall J versus multi-uniform-timeshifts d_2 versus d_3, for testing error at $O = 3n$ features. NRMSE; normalised root mean square error.

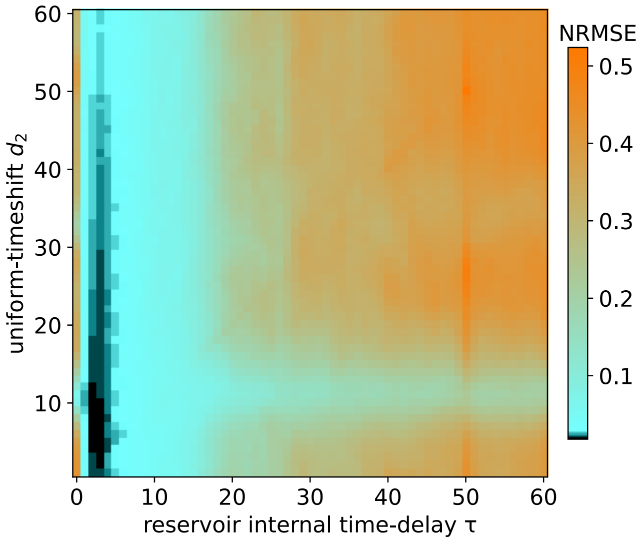

Suppl. Figure 7. Internal delay scan on the simulated reservoir computer, Mackey-Glass P-to-P 10-step-ahead forecasting. Scan of uniform-timeshift d_2 versus internal time-delay τ, for testing error at $O = 2n$ features at recall $J = 0$. NRMSE; normalised root mean square error.

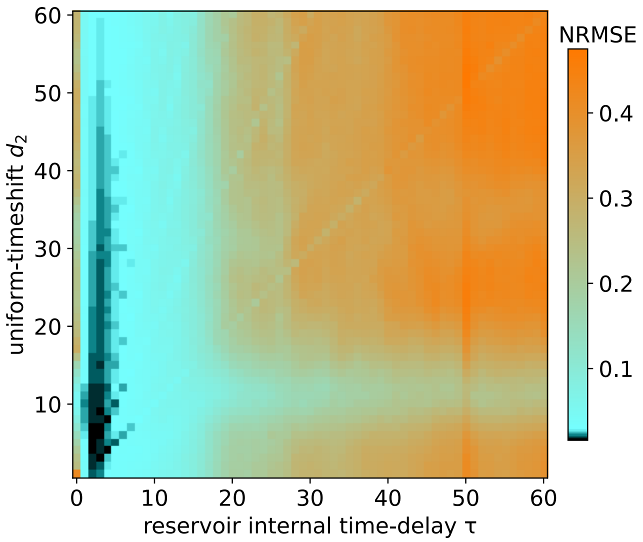

Suppl. Figure 8. Internal delay scan on the simulated reservoir computer, Mackey-Glass P-to-P 10-step-ahead forecasting. Scan of uniform-timeshift d_2 versus internal time-delay τ, for testing error at $O = 2n$ features at recall $J = n$. NRMSE; normalised root mean square error.

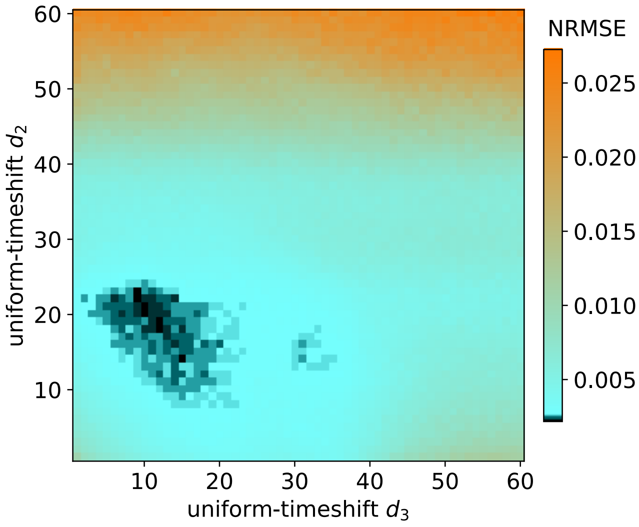

Suppl. Figure 9. Multi-uniform-timeshifting on the simulated reservoir computer, Rössler x-to-z cross-prediction. Scan of multi-uniform-timeshifts d_2 versus d_3, for testing error at $N = 3$ replicas and $O = 3n$ features. NRMSE; normalised root mean square error.

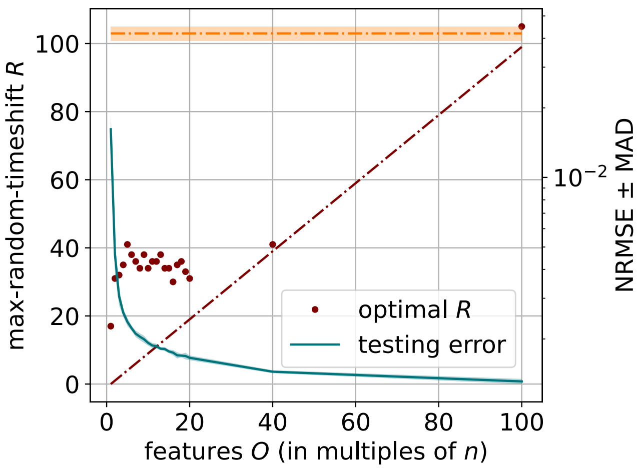

Suppl. Figure 10. Multi-random-timeshifting on the simulated reservoir computer, Rössler x-to-z cross-prediction. Scan of feature dimension O (up to $O = 100n$ features) versus max-random-timeshift R, for testing error (teal line). Maroon dots indicate optimal max-timeshift R at feature dimension O. Maroon dashed-line indicates the minimum value that the max-random-timeshift R may take at a given feature dimension. Orange dashed-line indicates result of training on the base state matrix $\boldsymbol{S}$. Shaded area around each line indicates the median absolute deviation of the realisation set. NRMSE; normalised root mean square error. MAD; median absolute deviation.

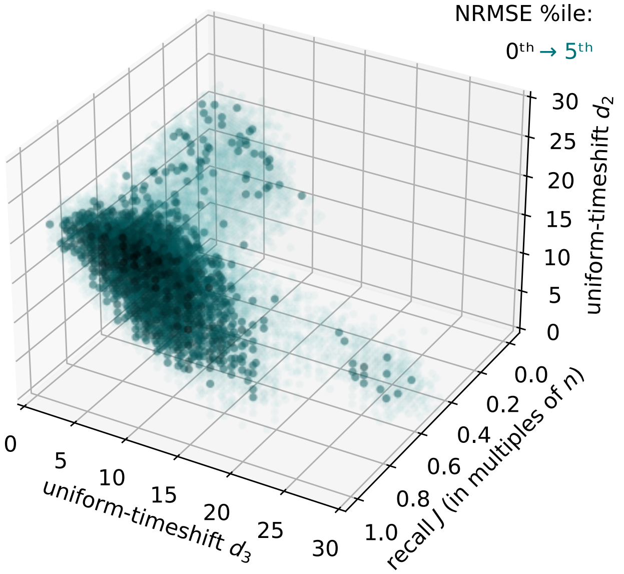

Suppl. Figure 11. Recall implementation on the simulated reservoir computer, Rössler x-to-z cross-prediction. Scan of recall J versus multi-uniform-timeshifts d_2 versus d_3, for testing error at $O = 3n$ features. NRMSE; normalised root mean square error.

Suppl. Table I. Multi-random-timeshifting ($\boldsymbol{S}_{r^1}{}^\frown...{}^\frown\boldsymbol{S}_{r^N}$) results for Lorenz x-to-z cross-prediction.

		clamped reservoir of n nodes, total recall $J = n$		rescaled reservoir of Nn nodes, null recall $J = 0$	
N replicas	O features	NRMSE ± MAD	R	NRMSE ± MAD	R
1	n	0.029 ± 0.005	19	0.028 ± 0.003	18
2	$2n$	0.011 ± 0.002	32	0.010 ± 0.002	33
3	$3n$	0.006 ± 0.001	40	0.0055 ± 0.0008	39
4	$4n$	0.0040 ± 0.0005	44	0.0037 ± 0.0009	43
5	$5n$	0.0028 ± 0.0007	57	0.0026 ± 0.0004	48
6	$6n$	0.0024 ± 0.0004	55	0.0021 ± 0.0003	51
10	$10n$	0.0014 ± 0.0002	59	0.0013 ± 0.0002	56
40	$40n$	0.00067 ± 0.00006	83	0.00062 ± 0.00004	71
100	$100n$	0.00045 ± 0.00003	100	0.00043 ± 0.00003	85

NRMSE; normalised root mean square error. MAD; median absolute deviation.

Suppl. Table II. Simulated reservoir computer results of Mackey-Glass P-to-P 10-step-ahead forecasting.

		uniform-timeshifting, $\boldsymbol{S}^\frown...{}^\frown\boldsymbol{S}_{d^\Sigma_N}$			random-timeshifting, $\boldsymbol{S}_{r^1}{}^\frown...{}^\frown\boldsymbol{S}_{r^N}$	
N	O	NRMSE	d_2	d_3	NRMSE ± MAD	R
1	n	*0.067 ± 0.005	-	-	0.032 ± 0.002	11
2	$2n$	0.022 ± 0.002	9	-	0.013 ± 0.001	13
3	$3n$	0.0109 ± 0.0006	6	4	0.0102 ± 0.0005	34
10	$10n$	-	-	-	0.0049 ± 0.0003	45
40	$40n$	-	-	-	0.0031 ± 0.0002	79
100	$100n$	-	-	-	0.0024 ± 0.0002	102

NRMSE; normalised root mean square error. MAD; median absolute deviation.
*Result of training on the base state matrix $\boldsymbol{S}$.

Suppl. Table III. Rössler attractor parameters.

$a = 0.2$	$b = 0.2$	$c = 5.7$	integration timestep d$t = 0.005$	signal discretision timestep = 0.1

Suppl. Table IV. Simulated reservoir computer results of Rössler x-to-z cross-prediction.

		uniform-timeshifting, $\boldsymbol{S}^\frown...{}^\frown\boldsymbol{S}_{d^\Sigma_N}$			random-timeshifting, $\boldsymbol{S}_{r^1}{}^\frown...{}^\frown\boldsymbol{S}_{r^N}$	
N	O	NRMSE	d_2	d_3	NRMSE ± MAD	R
1	n	*0.042 ± 0.003	-	-	0.016 ± 0.003	17
2	$2n$	0.0056 ± 0.0003	21	-	0.0046 ± 0.0008	31
3	$3n$	0.0022 ± 0.0002	22	9	0.0031 ± 0.0003	32
10	$10n$	-	-	-	0.00192 ± 0.00005	34
40	$40n$	-	-	-	0.00144 ± 0.00002	41
100	$100n$	-	-	-	0.00131 ± 0.00003	105

NRMSE; normalised root mean square error. MAD; median absolute deviation.
*Result of training on the base state matrix $\boldsymbol{S}$.

Suppl. Table V. Physical reservoir computer results of Mackey-Glass P-to-P 1-step-ahead forecasting.

		uniform-timeshifting, $\boldsymbol{S}^\frown...{}^\frown\boldsymbol{S}_{d^\Sigma_N}$						random-timeshifting, $\boldsymbol{S}_{r^1}{}^\frown...{}^\frown\boldsymbol{S}_{r^N}$	
N replicas	O features	NRMSE	d_2	d_3	d_4	d_5	d_6	NRMSE ± MAD	R
1	n	*0.231	-	-	-	-	-	0.088 ± 0.005	5
2	$2n$	0.059	5	-	-	-	-	0.068 ± 0.003	5
3	$3n$	0.047	4	1	-	-	-	0.060 ± 0.002	5
4	$4n$	0.046	3	1	1	-	-	0.054 ± 0.002	5
5	$5n$	0.045	3	1	1	11	-	0.051 ± 0.001	5
6	$6n$	0.045	1	3	1	2	2	0.047	5

NRMSE; normalised root mean square error. MAD; median absolute deviation.
*Result of training on the base state matrix $\boldsymbol{S}$.
